# The effect of zinc selenide nanospheres on porcine semen cryopreservation

**DOI:** 10.5713/ab.260067

**Published:** 2026-05-07

**Authors:** Bi Yu Zhang, Hai Dong Liu, Fu Qiang Chang, Ju Meng Wei, Jing Li, Wen Chao Li, Chong Mei Ruan

**Affiliations:** 1 College of Animal Science, Anhui Science and Technology University, Fengyang, China; 2 College of Chemistry and Materials Engineering, Anhui Science and Technology University, Bengbu, China; 3 Anhui Province Key Laboratory of Animal Nutrition Regulation and Health, Fengyang, China; 4 Anhui Engineering Research Center of Pork Quality Control and Enhancement, Fengyang, China

**Keywords:** Nanospheres, Porcine Semen Cryopreservation, Quality of Sperm, Zinc Selenide

## Abstract

**Objective:**

This study aimed to investigate the protective effects of zinc selenide (ZnSe) nanospheres on boar semen during cryopreservation and to determine the optimal supplementation concentration for improving post-thaw sperm quality and antioxidant status.

**Methods:**

ZnSe nanospheres were synthesized via a hydrothermal method using Na_2_SeO_3_ and Zn(CH_3_COO)_2_ as precursors. Ejaculates with motility ≥70% were collected from six healthy boars, pooled to minimize individual variation, and the entire experiment was independently repeated three times. Samples were mixed with cryopreservation extenders containing 0 (control), 0.1, 1, or 10 mmol/L ZnSe nanospheres. Post-freezing and thawing, sperm motility parameters, acrosome integrity, plasma membrane integrity, antioxidant enzyme activities, and intracellular reactive oxygen species (ROS) levels were evaluated.

**Results:**

Supplementation with 1 mmol/L ZnSe significantly improved post-thaw sperm motility, acrosome integrity, and plasma membrane integrity (p<0.05). Moreover, 1 mmol/L ZnSe markedly enhanced total antioxidant capacity, catalase, and superoxide dismutase activities, while reducing malondialdehyde concentrations and intracellular ROS levels (p<0.05).

**Conclusion:**

Supplementation with 1 mmol/L ZnSe in the cryopreservation diluent effectively improves post-thaw sperm quality and antioxidant capacity, offering a promising strategy for optimizing porcine semen cryopreservation.

## INTRODUCTION

Semen cryopreservation is integral to the widespread adoption of artificial insemination, the effective utilization and genetic enhancement of boar populations, and the overall improvement of reproductive outcomes in pig breeding systems [1]. However, during the freezing process, the fluidity of the phospholipid bilayer of the sperm plasma membrane decreases markedly [2], leading to impaired membrane integrity, abnormal permeability [3], disruption of intracellular ion homeostasis, and excessive accumulation of reactive oxygen species (ROS) [4]. Such disturbances exacerbate cryodamage, manifested as reduced motility and viability, compromised plasma membrane integrity, mitochondrial dysfunction, and DNA fragmentation [5]. The use of antioxidants to alleviate oxidative stress during semen cryopreservation has become an important strategy for improving sperm quality after freeze-thaw cycles. To mitigate oxidative stress during cryopreservation, a variety of antioxidants—such as vitamin E, glutathione, resveratrol, and selenium-containing compounds—have been evaluated and shown to partially improve post-thaw sperm quality [6]. Nonetheless, traditional antioxidants exhibit inherent limitations, including poor stability, rapid degradation, limited bioavailability, and insufficient sustained-release properties, all of which restrict their protective effects during the freeze–thaw process. As a result, there is an increasing interest in the development of novel antioxidant systems that offer enhanced stability, superior free radical-scavenging capabilities, and extended biological activity, with particular emphasis on nanomaterial-based antioxidants.

Zinc selenide (ZnSe) nanoparticles, a class of II–VI semiconductor nanomaterials, have demonstrated favorable biocompatibility under controlled conditions [7]. In recent years, their potential applications in biomedical imaging, drug delivery, and cytoprotection have been increasingly reported [8], including their beneficial effects on germ cells [9]. ZnSe nanoparticles possess uniform particle size distribution, large specific surface area, and unique crystallographic features [10], enabling efficient electron transfer [11] and strong free radical-scavenging activity. As a metal selenide, ZnSe can effectively remove excess intracellular ROS and modulate inflammation by inhibiting the NF-κB signaling pathway, thereby reducing the expression of tumor necrosis factor-α (TNF-α) and interleukin-6 (IL-6) [12]. However, despite these advantages, limited studies have examined the application of ZnSe nanoparticles in the field of porcine semen cryopreservation. Therefore, the present study aimed to evaluate the protective role of ZnSe nanospheres in mitigating cryodamage, improving sperm functional parameters, and enhancing antioxidant capacity during the freeze–thaw process. This research provides a foundation for developing novel antioxidant strategies for porcine semen cryopreservation and potentially for broader applications in livestock reproductive biotechnology.

## MATERIALS AND METHODS

Semen samples were collected from six healthy adult Large White boars (2–3 years old, 180–220 kg). To minimize individual variation, semen samples collected from six healthy boars were pooled to generate one biological replicate. The entire experimental procedure, including semen collection, pooling, dilution, cryopreservation, thawing, and subsequent analyses, was independently repeated three times using freshly collected semen samples. Therefore, a total of three biological replicates (n = 3; each replicate represents pooled semen from six boars) were included in this study. Within each biological replicate, all measurements were performed in triplicate as technical replicates. All boars exhibited normal libido and had a documented history of good semen quality based on routine monitoring. Animals were housed at Shili Breeding Pig Company in Fengyang County, Anhui Province, under controlled environmental conditions (temperature 18°C–22°C; relative humidity 50%–60%; 12 h light/dark cycle) with free access to water and a commercial breeding diet formulated according to NRC (2012) standards. Semen was collected using the hand-glove method and transported to the laboratory within 30 min of ejaculation. Ejaculates with sperm motility ≥0.7 and sperm concentration ≥3×10^8^ cells/mL were selected for further analyses.

### Test materials

Unless otherwise specified, all reagents used in this study were of analytical grade and purchased from Sigma-Aldrich. Coomassie Brilliant Blue G-250 was obtained from Shanghai Sangon Biotech. The ROS assay kit was purchased from Jiangsu Kaiji Biotechnology, while the assay kits for superoxide dismutase (SOD), malondialdehyde (MDA), total antioxidant capacity (T-AOC), and catalase (CAT) were all supplied by Nanjing Jiancheng Bioengineering Institute.

The major instruments used in this study included a phase-contrast microscope (AE2000; Macody), a microplate reader (Multiskan GO; Thermo Fisher Scientific), a scanning electron microscope (SEM, JSM-IT810; JEOL), an X-ray diffractometer (D8 Advance; Bruker), a fully automated sperm analyzer (ML-608JZ; Nanning Songjing Tianlun Biotechnology), and an upright fluorescence microscope (BX63; Olympus).

### Preparation of zinc selenide nanospheres

**Figure 1:**
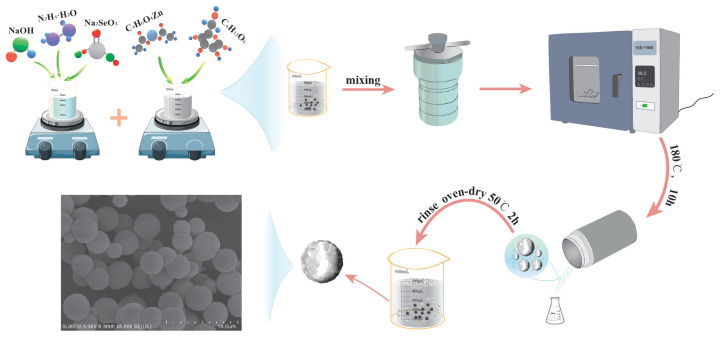
Schematic diagram of ZnSe nanospheres preparation process. Technique summary: ZnSe nanospheres were synthesized using a hydrothermal method. Na_2_SeO_3_ and Zn(CH_3_COO)_2_ were used as precursors in the presence of hydrazine hydrate and glucose. The reaction was carried out at 180°C for 10 h in a Teflon-lined autoclave. After natural cooling, the product was washed with ethanol and deionized water, and dried at 50°C for 2 h to obtain ZnSe nanosphere powder. ZnSe, zinc selenide.

ZnSe nanospheres were synthesized using a hydrothermal method with slight modifications based on the procedure described by Duan et al. [13]. Briefly, Na_2_SeO_3_ (2 mmol) and NaOH (0.1 mol) were dissolved in 10 mL of deionized water, followed by sonication and stirring until a clear homogeneous solution was obtained. Hydrazine hydrate (3.4 mL) was then added, and stirring was continued. Subsequently, Zn(CH_3_COO)_2_ (2 mmol) and glucose (0.5 g) were introduced into the mixture, which was again sonicated and stirred thoroughly. The resulting solution was transferred into a 48 mL Teflon-lined stainless-steel autoclave and filled to approximately 75% of its volume with deionized water. The sealed reactor was heated at 180°C for 10 h. After naturally cooling to room temperature, the product was collected, washed several times with ethanol and deionized water, and dried at 50°C for 2 h to obtain ZnSe nanospheres powder (Figure 1).

### Characterization validation

The morphology of the ZnSe particles was characterized following the criteria described by Lambe and Brady [14]. The morphology and approximate size of ZnSe nanospheres were characterized by SEM, which revealed relatively uniform spherical particles. The crystalline phase of the product was analyzed using X-ray diffraction (XRD) over a 2θ range of 20°–80° with a step size of 0.02°, and the diffraction pattern was compared with the standard JCPDS card No. 37-1463 for phase identification.

### Experimental design and processing

#### Diluent preparation and experimental design

The basic extender for boar semen freezing consisted of 3.3 g glucose, 4.44 g citric acid, 7.26 g Tris (trihydroxymethyl aminomethane), and 0.18 g penicillin–streptomycin, diluted to a final volume of 300 mL with distilled water.

The cryopreservation extender was prepared by mixing 60 mL of egg yolk and 9 mL of glycerol with the basic extender and adjusting the final volume to 300 mL, followed by thorough stirring. The extender was freshly prepared prior to use.

ZnSe nanospheres powder was ultrasonically dispersed in the cryopreservation extender for 10 min (200 W, 3 s on / 5 s off) to obtain ZnSe-containing extenders with final concentrations of 0 mmol/L (control), 0.1 mmol/L, 1 mmol/L, 10 mmol/L. The pooled semen sample was divided into four treatment groups corresponding to different ZnSe concentrations.

#### Semen freezing and thawing

Selected boar semen samples that met the quality criteria were processed for cryopreservation. Seminal plasma was removed by centrifugation at 800×g for 10 min at room temperature. The resulting sperm pellets were resuspended in the prepared cryopreservation extender at a ratio of 1:1 (v/v). The extender contained different concentrations of ZnSe nanospheres (0, 0.1, 1, and 10 mmol/L).

The diluted semen samples were gently mixed and loaded into 0.5 mL plastic straws, which were then sealed and equilibrated at 4°C for 90 min. After equilibration, the straws were horizontally placed 3 cm above the surface of liquid nitrogen for 10 min to allow controlled cooling in nitrogen vapor, followed by direct immersion into liquid nitrogen (−196°C) for long-term storage.

For thawing, the straws were removed from liquid nitrogen and immediately immersed in a 37°C water bath for 30 s. The thawed semen was then diluted with prewarmed physiological saline (37°C) to obtain a final sperm concentration of 1×10^7^ sperm/mL. Subsequently, the samples were subjected to further analyses, including sperm motility assessment, acrosome and plasma membrane integrity evaluation, and determination of antioxidant enzyme activities (SOD, CAT, and T-AOC) as well as MDA content.

### Detection indicators and methods

#### Sperm motility parameters

A 10 μL aliquot of semen was placed on a pre-warmed (37°C) Leja slide (MLCASA; Mailang). Sperm motility parameters, including total motility, progressive motility, straightline velocity (VSL), curvilinear velocity (VCL), and average path velocity (VAP), were evaluated using a computer-assisted sperm analysis (CASA) system. At least five randomly selected microscopic fields were analyzed for each sample, and a minimum of 200 spermatozoa were evaluated per field.

#### Sperm acrosome integrity testing

Sperm acrosome integrity was assessed using Coomassie Brilliant Blue staining, following a slightly modified protocol described by Larson and Miller [15]. Briefly, 50 μL of semen was mixed with 1 mL of 4% paraformaldehyde in a centrifuge tube and fixed for 10 min with gentle agitation. The sample was then centrifuged at 424×g for 3 min, the supernatant was discarded, and the pellet was resuspended in phosphate-buffered saline (PBS). A 10 μL aliquot of the fixed semen was used to prepare a smear, which was air-dried, stained with Coomassie Brilliant Blue for 30 min, rinsed with water, and air-dried again prior to microscopic examination. Sperm with a clearly stained acrosomal region were classified as having intact acrosomes, whereas those lacking staining in this region were considered to have damaged or incomplete acrosomes. At least five microscopic fields were examined per sample, and a minimum of 200 sperm were counted in each field to calculate the acrosome integrity rate.

#### Sperm plasma membrane integrity detection

Sperm plasma membrane integrity was assessed using a commercially available sperm viability kit, according to the manufacturer’s instructions. Mix 100 μL of semen with 1 mL of reagent solution thoroughly, incubate at 37°C for 1 h, then take 10 μL of the sample onto a glass slide and observe under a phase-contrast microscope (400×). Sperm with intact plasma membranes show tail curvature or swelling. At least five fields of view were examined under a microscope, with a minimum of 200 sperm counted per field. The integrity rate of the sperm plasma membrane was then calculated.

#### Sperm antioxidant index testing

Antioxidant enzyme levels in thawed porcine semen were measured using commercially available assay kits for SOD, MDA, T-AOC, and CAT, according to the manufacturer’s instructions. Each sample was tested in triplicate using a microplate reader, and the results were subjected to statistical analysis.

#### Sperm reactive oxygen species content detection

Intracellular ROS levels were measured using a commercially available ROS detection kit according to the manufacturer’s instructions. Briefly, thawed semen samples were incubated with the fluorescent probe at 37°C for 20 min in the dark. For the positive control (PC) group, thawed sperm samples were treated with 100 μM hydrogen peroxide (H_2_O_2_) at 37°C for 30 min to induce oxidative stress prior to staining. After incubation, the samples were washed with PBS to remove excess dye and immediately observed under a fluorescence microscope.

Fluorescence images were captured using identical exposure settings for all samples. The fluorescence intensity, which reflects intracellular ROS levels, was quantified using ImageJ 1.8.0 software. At least five randomly selected fields were analyzed per sample, and a minimum of 200 spermatozoa were evaluated for each group. The average fluorescence intensity was calculated and used for statistical analysis.

### Statistical analysis

Statistical analysis was performed using SPSS ver. 26.0. Data are presented as the mean±standard deviation (mean±SD) of three independent biological replicates (n = 3; each replicate represents pooled semen from six boars). Within each biological replicate, all measurements were performed in triplicate, and the averaged values were used for statistical analysis. Differences among groups were analyzed using one-way analysis of variance (ANOVA) followed by Tukey’s multiple comparison test, and statistical significance was defined as p<0.05.

Graphs were generated using GraphPad Prism version 9.0. Fluorescence images were analyzed using ImageJ software, while SEM images and XRD patterns of ZnSe nanoparticles were processed and plotted using Origin 2023 software.

## RESULTS

### Crystal structure and morphology characterization results of zinc selenide

**Figure 2:**
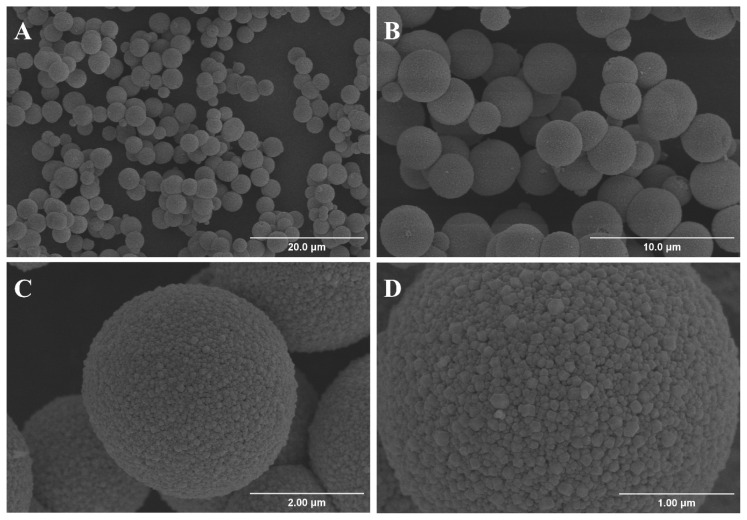
Morphology and microstructure of ZnSe nanospheres observed by scanning electron microscopy (SEM). Technique summary: The synthesized ZnSe nanospheres were sputter-coated with gold and imaged using a JSM-IT810 scanning electron microscope (JEOL) at an accelerating voltage of 15 kV. Images (A–D) are shown at increasing magnifications (scale bars: 20.0 μm, 10.0 μm, 2.00 μm, and 1.00 μm, respectively) to illustrate particle shape, size distribution, and surface substructure. ZnSe, zinc selenide.

**Figure 3:**
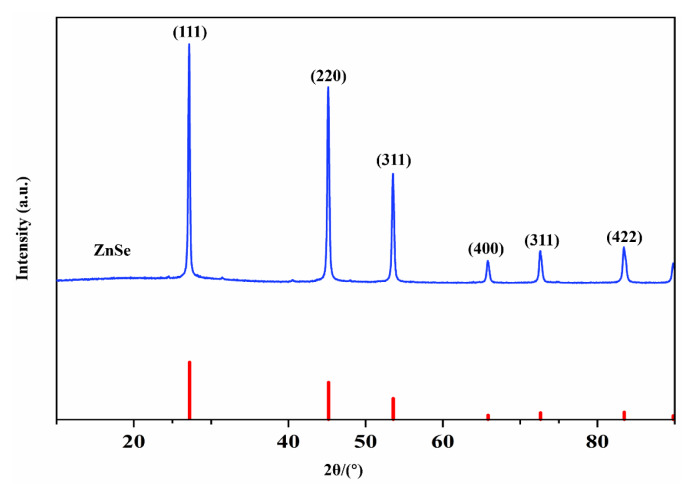
X-ray diffraction (XRD) pattern of ZnSe nanospheres. Technique summary: XRD analysis was performed using a D8 Advance X-ray diffractometer (Bruker) with Cu-Kα radiation (λ = 1.5406 Å). Data were collected over a 2θ range of 20°–80° at a step size of 0.02°. The three major diffraction peaks at 2θ = 27.50°, 45.60°, and 54.10° correspond to the (111), (220), and (311) crystal planes, respectively, and are consistent with the standard ZnSe pattern (JCPDS card No. 37-1463), confirming the phase purity of the product. ZnSe, zinc selenide.

Figures 2A–2D show that the synthesized ZnSe nanoparticles particles exhibit a predominantly spherical morphology with a relatively uniform particle size distribution. At higher magnifications (Figures 2C, 2D), the particle surfaces are observed to be composed of smaller subunits, indicating a hierarchical structural organization. As shown in Figure 3, the sample displays three prominent diffraction peaks at 2θ = 27.50°, 45.60°, and 54.10°, corresponding to the (111), (220), and (311) crystal planes, respectively. These peaks are consistent with the standard ZnSe reference pattern (JCPDS No. 37-1463), confirming the successful synthesis of ZnSe. Additionally, the absence of impurity peaks in the XRD spectrum suggests that the obtained product possesses high phase purity.

### Effects of zinc selenide on boar sperm motility parameters

As shown in Table 1, both sperm motility and viability were significantly higher in the ZnSe-treated groups than in the control group (p<0.05), with the 1 mmol/L group exhibiting the highest values (p<0.05). The VSL of the 1 mmol/L group was also significantly higher than that of the other treatment groups (p<0.05), whereas the 0.1 mmol/L group showed a slight, nonsignificant increase in VSL compared with the control and 10 mmol/L groups (p>0.05). The VCL of the 1 mmol/L group showed a slight improvement relative to the control group, although the difference was not statistically significant (p>0.05). In contrast, VAP was significantly higher in the 1 mmol/L group than in all other groups (p<0.05).

### Effects of zinc selenide on the integrity of porcine sperm acrosome and plasma membrane

**Figure 4:**
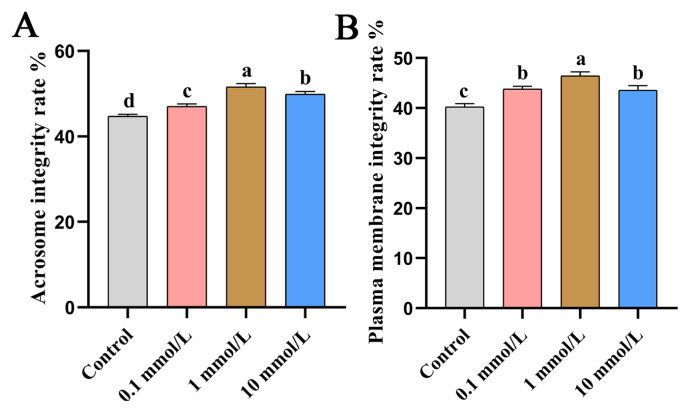
Effects of ZnSe nanospheres on acrosome integrity and plasma membrane integrity in porcine sperm. Technique summary: (A) Acrosome integrity was assessed using Coomassie Brilliant Blue staining. After fixation with 4% paraformaldehyde, smears were stained for 30 min. Sperm with a clearly stained acrosomal region were counted as intact under light microscopy (400×). (B) Plasma membrane integrity was evaluated using a hypo-osmotic swelling test. Sperm showing tail curvature or swelling were considered to have intact membranes. For each sample, at least five microscopic fields and a minimum of 200 sperm per field were examined. Data are presented as mean±SD from three independent biological replicates (n = 3). ^a–d^ Different lowercase letters indicate p<0.05, while the same letter or no letter indicates p>0.05, one-way ANOVA followed by Tukey’s test. ZnSe, zinc selenide; SD, standard deviation; ANOVA, analysis of variance.

As shown in Figure 4A, acrosome integrity was improved in all ZnSe-treated groups, with the 1 mmol/L group exhibiting the highest integrity rate, significantly higher than those of the other groups (p<0.05). Similarly, Figure 4B shows that plasma membrane integrity was enhanced in all treatment groups compared with the control, and the 1 mmol/L group displayed the highest rate, also significantly higher than that of the other groups (p<0.05). No significant difference was observed between the 0.1 mmol/L and 10 mmol/L groups (p>0.05).

### Effects of zinc selenide on the content of antioxidant enzymes in porcine sperm

**Figure 5:**
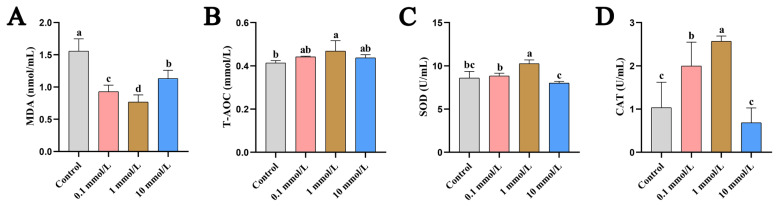
Effect of ZnSe nanospheres on antioxidant parameters in porcine sperm. Technique summary: After thawing, sperm samples were analyzed for (A) malondialdehyde (MDA) content, (B) total antioxidant capacity (T-AOC), (C) superoxide dismutase (SOD) activity, and (D) catalase (CAT) activity using commercial colorimetric assay kits (Nanjing Jiancheng Bioengineering Institute). Absorbance was measured using a Multiskan GO microplate reader (Thermo Fisher Scientific). All assays were performed in triplicate within each biological replicate. Data are presented as mean±SD from three independent biological replicates (n = 3). ^a–d^ Different lowercase letters indicate p<0.05, while the same letter or no letter indicates p>0.05, one-way ANOVA followed by Tukey’s test. ZnSe, zinc selenide; SD, standard deviation; ANOVA, analysis of variance.

As shown in Figure 5A, the MDA content was significantly reduced in all ZnSe-treated groups compared with the control group (p<0.05), with the lowest level observed in the 1 mmol/L group. Figure 5B indicates that T-AOC was significantly higher in the 1 mmol/L group than in the control group (p<0.05), while the 0.1 mmol/L and 10 mmol/L groups showed slight but nonsignificant increases.

In Figure 5C, SOD activity was significantly higher in the 1 mmol/L group than in the control, 0.1 mmol/L, and 10 mmol/L groups (p<0.05). As shown in Figure 5D, CAT activity was also highest in the 1 mmol/L group, significantly exceeding that of the other groups (p<0.05). Although CAT activity in the 10 mmol/L group was lower than that of the control group, the difference was not statistically significant (p>0.05).

### Effect of zinc selenide on reactive oxygen species content in porcine sperm

**Figure 6:**
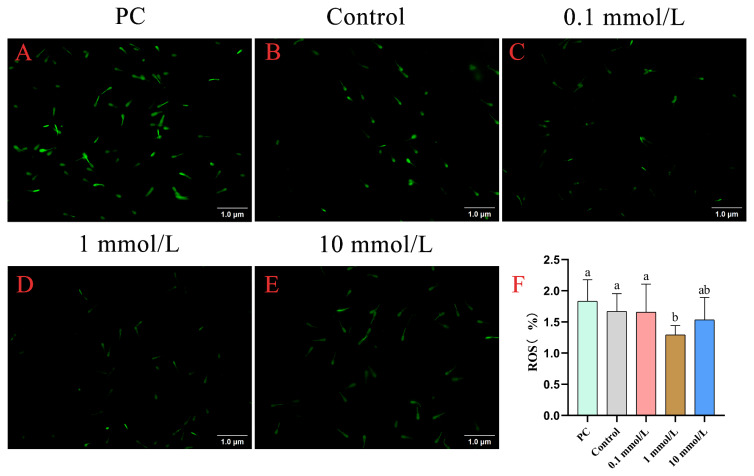
Effect of ZnSe nanospheres on reactive oxygen species (ROS) levels in porcine sperm. Technique summary: Intracellular ROS levels were detected using the fluorescent probe DCFH-DA. Briefly, frozen sperm were thawed and incubated with DCFH-DA staining solution at 37 °C for 20 min under dark conditions. The positive control (PC) group was pretreated with 100 μM H_2_O_2_ at 37°C for 30 min before DCFH-DA staining to induce cellular oxidative stress. After incubation, the sperm were washed to remove unbound probe. Fluorescence images (A–E) were captured using a BX63 upright fluorescence microscope (Olympus) under identical exposure settings. (F) Quantitative analysis: mean fluorescence intensity was measured from at least five randomly selected fields per sample (≥200 sperm per field) using ImageJ 1.8.0 software. Data are presented as mean±SD from three independent biological replicates (n = 3). ^a,b^ Different lowercase letters indicate p<0.05, while the same letter or no letter indicates p>0.05, one-way ANOVA followed by Tukey’s test. ZnSe, zinc selenide; SD, standard deviation; ANOVA, analysis of variance.

Figure 6 shows the fluorescence images of the PC, control, 0.1 mmol/L, 1 mmol/L, and 10 mmol/L groups. The PC group exhibited the strongest fluorescence intensity, confirming successful induction of oxidative stress. Among these, the 1 mmol/L group exhibited the weakest fluorescence intensity. As shown in Figure 6F, ROS levels were significantly lower in the 1 mmol/L group than in the control group (p<0.05). Although the 10 mmol/L group also showed a reduction in ROS levels, the difference was not statistically significant (p>0.05).

## DISCUSSION

Previous studies have demonstrated that ZnSe nanomaterials possess favorable biocompatibility in various *in vitro* models, exhibiting low cytotoxicity toward HEK cells at appropriate doses [12] and enhanced biological activity following oleic acid modification [16]. In addition, plant-derived ZnSe nanoparticles have been reported to show acceptable cytocompatibility [17], further supporting their potential biosafety. Building on these findings, the present study evaluated the applicability of ZnSe in a boar semen cryopreservation system. The results showed that, at suitable concentrations, ZnSe supplementation did not exert detrimental effects on sperm; instead, it enhanced post-thaw sperm motility, plasma membrane integrity, and antioxidant capacity. These findings indicate that ZnSe nanomaterials may serve as a promising cryoprotective additive for improving the freeze–thaw resilience of boar sperm.

During cryopreservation, sperm are exposed to a variety of stressors, such as osmotic imbalance, ice crystal formation, cold shock, and oxidative damage, all of which disrupt cellular homeostasis [18]. These stress responses reduce the fluidity of the phospholipid bilayer, alter membrane permeability [19], and impair mitochondrial function, ultimately leading to excessive accumulation of reactive ROS [20] and a decline in sperm motility and fertilizing potential. Although traditional antioxidants can mitigate some aspects of cryodamage, their physicochemical instability and the extreme conditions of the freeze–thaw process often limit their effectiveness. For instance, conventional antioxidant strategies may fail to provide sustained protection under cryogenic conditions, thereby limiting their capacity to effectively scavenge ROS and maintain sperm function [21], whereas recent studies have demonstrated that advanced cryoprotective approaches, including growth factor-enriched or optimized systems, can significantly improve post-thaw sperm quality by enhancing cellular resilience to oxidative stress [22]. At the same time, it is expected that the combined use of multiple cryoprotectants can provide synergistic protection, but in the cryopreservation of boar sperm, it may instead increase cytotoxicity and osmotic stress, complicate the optimization of the protocol, reduce reproducibility, and ultimately impair the overall cryopreservation survival rate [23]. Nanomaterials offer potential advantages in semen cryopreservation due to their large specific surface area, unique physicochemical properties, and sustained-release capacity [24]. In particular, ZnSe nanoparticles exhibit notable antioxidant and anti-inflammatory properties, positioning them as promising alternatives to traditional antioxidants. Previous studies reported that ZnSe quantum dots significantly enhanced CAT and peroxidase activities in maize leaves while reducing hydrogen peroxide levels [25], and that ZnSe nanoparticle-based systems can enhance antioxidant enzyme activities and reduce oxidative damage in biological systems [26]. In this study, we investigated the optimal concentration of ZnSe nanospheres for boar semen cryopreservation. Supple-mentation with 1 mmol/L ZnSe nanospheres produced the most beneficial effects, yielding the highest levels of sperm viability, motility, and velocity parameters. These improvements may be attributed to the ability of ZnSe nanospheres to alleviate ROS-induced mitochondrial damage, maintain mitochondrial function, and support sustained ATP production, thereby enhancing the post-thaw functional quality of sperm [27].

The sperm plasma membrane serves as the primary interface between the sperm cell and its external environment. By regulating the transmembrane transport of ions and metabolites, it maintains intracellular homeostasis and membrane potential balance, thereby supporting essential reproductive processes such as capacitation, hyperactivated motility, and the acrosome reaction [28]. In the present study, the 1 mmol/L ZnSe group exhibited significantly higher plasma membrane integrity than the other groups. Similar findings have been reported in goat sperm, where supplementation with ZnO and Se nanoparticles during cryopreservation enhanced antioxidant enzyme activity after thawing [29]. This improvement may be attributed to the ability of ZnSe nanospheres to increase CAT activity, limit lipid peroxidation, and stabilize mem-brane permeability [26]. Acrosome integrity, a crucial indicator of sperm fertilization potential, was also improved in all ZnSe-treated groups, with the greatest enhancement observed in the 1 mmol/L group. Previous studies have likewise shown that supple-mentation with zinc oxide or selenium oxide nanoparticles improved acrosome integrity in rat sperm following cryopreservation [30]. Zhang et al. [26] further demonstrated that incorporating zinc oxide nanoparticles into cryopreservation media enhanced sperm motility and antioxidant enzyme activity. In equine sperm, the addition of zinc nanoparticles to cryoprotective extenders similarly improved plasma membrane integrity and overall membrane stability [31]. Collectively, these findings support the protective role of metal-based nanomaterials in mitigating cryodamage and highlight ZnSe nanospheres as a promising candidate for improving boar sperm cryopreservation outcomes.

During oxidative phosphorylation, electron leakage from the mitochondrial respiratory chain leads to the generation of reactive ROS. Although physiological levels of ROS are essential for processes such as sperm capacitation, the acrosome reaction, fertilization competence, and sperm–oocyte interaction, excessive ROS can induce oxidative damage and impair sperm function [32]. Increasing intracellular antioxidant enzyme activity is an effective strategy for limiting ROS overproduction, thereby reducing lipid peroxidation and improving overall semen quality. Recent studies have further demonstrated that enhancing endogenous antioxidant defense systems is a key approach to improving sperm cryotolerance and maintaining post-thaw functional integrity [33]. In the present study, ZnSe nanoparticles were utilized as an exogenous antioxidant source to enhance sperm protection during cryopreservation. Selenium, a critical component of glutathione peroxidase, plays a central role in maintaining membrane stability and preventing oxidative damage by scavenging ROS [34]. Zinc, as the structural metal of Cu/Zn-SOD, stabilizes the enzyme’s conformation and catalytic efficiency, thereby promoting SOD-mediated dismutation of superoxide anions and reducing intracellular ROS accumulation. Adequate zinc levels in seminal plasma can additionally improve sperm function by enhancing SOD activity, inhibiting lipid peroxidation, and maintaining chromatin and membrane stability [35]. Previous studies have shown that supple-mentation with selenium oxide nanoparticles during cryopreservation reduced MDA levels in rat sperm post-thaw, consistent with the present findings. This effect may be attributed to the ability of ZnSe nanoparticles to enhance SOD and glutathione peroxidase activities, ultimately reducing MDA production and limiting lipid peroxidation. In our study, ZnSe nanospheres significantly improved T-AOC, CAT, and SOD activities, with the strongest antioxidant response observed at the 1 mmol/L concentration. Similarly, Heidari et al. [36] reported that zinc oxide nanoparticle supplementation reduced MDA levels in thawed ram semen, further supporting the antioxidant efficacy of metal-based nanomaterials in mitigating cryodamage.

ROS are generated through the mitochondrial electron transport chain and function as signaling molecules involved in cellular metabolism and immune regulation. However, excessive accumulation of ROS can induce oxidative damage, mitochondrial dysfunction, and even DNA mutations [37]. Nanomaterial-based antioxidant strategies have recently been recognized as effective approaches to regulate ROS homeostasis and improve cellular resistance to oxidative stress [38]. Piri et al. [39] reported that selenium nanoparticles effectively reduced ROS levels in cryopreserved goat sperm. In the present study, supplementation with ZnSe nanoparticles markedly reduced ROS content in boar sperm following thawing, with the strongest effect observed at the 1 mmol/L concentration. This reduction may be attributed to the ability of ZnSe nanoparticles to scavenge excessive ROS and suppress the lipid peroxidation cascade, thereby enhancing the resilience of sperm to freeze–thaw-induced oxidative stress. These findings are in agreement with those reported by Isaac et al. [40], further supporting the antioxidative potential of ZnSe-based nanomaterials in semen cryopreservation.

## CONCLUSION

However, the present study is limited by the absence of in vivo fertility evaluation, and future studies are required to assess the reproductive outcomes following artificial insemination. In addition, although functional and biochemical indicators were comprehensively evaluated, ultrastructural observations such as SEM were not performed to directly visualize sperm morphological alterations after cryopreservation. Future investigations incorporating SEM analysis would provide further insight into the structural changes associated with ZnSe supplementation.

## Figures and Tables

**Table 1:** Effects of ZnSe nanospheres on motility parameters of boar sperm

Item	Control	0.1 mmol/L	1 mmol/L	10 mmol/L
Sperm viability (%)	29.96±0.50^c^	33.13±0.43^b^	38.97±0.62^a^	33.40±0.70^b^
Progressive motility (%)	27.51±0.85^c^	30.92±0.58^b^	35.37±0.54^a^	31.02±0.51^b^
VSL (μm/s)	20.42±0.26^c^	23.66±0.45^b^	27.31±1.94^a^	15.42±1.73^d^
VCL (μm/s)	46.24±0.47^a^	37.63±1.01^b^	48.02±6.53^a^	18.26±0.50^c^
VAP (μm/s)	32.86±0.40^b^	25.43±0.59^c^	38.05±2.77^a^	17.98±2.41^d^

Technique summary: After thawing, sperm motility parameters were evaluated using a computer-assisted sperm analysis (CASA) system (ML-608JZ; Nanning Songjing Tianlun Biotechnology).

Parameters assessed included sperm viability (%), progressive motility (%), straight-line velocity (VSL, μm/s), curvilinear velocity (VCL, μm/s), and average path velocity (VAP, μm/s).

Data are presented as mean±SD from three independent biological replicates (n = 3; each replicate represents pooled semen from six boars).

a–d Different lowercase letters indicate p<0.05, while the same letter or no letter indicates p>0.05, one-way ANOVA followed by Tukey’s post hoc test.

ZnSe, zinc selenide; SD, standard deviation; ANOVA, analysis of variance.

## Data Availability

Upon reasonable request, the datasets of this study can be available from the corresponding author.
